# Patient understanding regarding opioid use in an orthopaedic trauma surgery population: a survey study

**DOI:** 10.1186/s13018-021-02881-w

**Published:** 2021-12-24

**Authors:** Amy L. Xu, Alexandra M. Dunham, Zachary O. Enumah, Casey J. Humbyrd

**Affiliations:** 1 Department of Orthopaedic Surgery, Johns Hopkins Hospital, Baltimore, MD USA; 2 Department of Surgery, Johns Hopkins Hospital, Baltimore, MD USA; 3 Department of Orthopaedic Surgery, The University of Pennsylvania, 230 West Washington Square, 5th Floor Farm Journal Building,, Philadelphia, PA 19106 USA

**Keywords:** Opioid misuse, Patient knowledge, Addiction, Dependence, Naloxone

## Abstract

**Background:**

Prior studies have assessed provider knowledge and factors associated with opioid misuse; similar studies evaluating patient knowledge are lacking. The purpose of this study was to assess the degree of understanding regarding opioid use in orthopaedic trauma patients. We also sought to determine the demographic factors and clinical and personal experiences associated with level of understanding.

**Methods:**

One hundred and sixty-six adult orthopaedic trauma surgery patients across two clinical sites of an academic institution participated in an internet-based survey (2352 invited, 7.1% response rate). Demographic, clinical, and personal experience variables, as well as perceptions surrounding opioid use were collected. Relationships between patient characteristics and opioid perceptions were identified using univariate and multivariable logistic regressions. Alpha = 0.05.

**Results:**

Excellent recognition (> 85% correct) of common opioids, side effects, withdrawal symptoms, and disposal methods was demonstrated by 29%, 10%, 30%, and 2.4% of patients; poor recognition (< 55%) by 11%, 56%, 33%, and 52% of patients, respectively. Compared with white patients, non-white patients had 7.8 times greater odds (95% confidence interval [CI] 1.9–31) of perceiving addiction discrepancy (*p* = 0.004). Employed patients with higher education levels were less likely to have excellent understanding of side effects (adjusted odds ratio [aOR] 0.06, 95% CI 0.006–0.56; *p* = 0.01) and to understand that dependence can occur within 2 weeks (aOR 0.28, 95% CI 0.09–0.86; *p* = 0.03) than unemployed patients. Patients in the second least disadvantaged ADI quartile were more knowledgeable about side effects (aOR 8.8, 95% CI 1.7–46) and withdrawal symptoms (aOR 2.7, 95% CI 1.0–7.2; *p* = 0.046) than those in the least disadvantaged quartile. Patients who knew someone who was dependent or overdosed on opioids were less likely to perceive addiction discrepancy (aOR 0.24, 95% CI 0.07–0.76; *p* = 0.02) as well as more likely to have excellent knowledge of withdrawal symptoms (aOR 2.6, 95% CI 1.1–6.5, *p* = 0.03) and to understand that dependence can develop within 2 weeks (aOR 3.8, 95% CI 1.5–9.8, *p* = 0.005).

**Conclusions:**

Level of understanding regarding opioid use is low among orthopaedic trauma surgery patients. Clinical and personal experiences with opioids, in addition to demographics, should be emphasized in the clinical history.

**Supplementary Information:**

The online version contains supplementary material available at 10.1186/s13018-021-02881-w.

## Background

The United States has been in the midst of a public health crisis—nearly 70% of the 67,367 drug overdose deaths in 2018 reported involvement of an opioid [1]. Prescription opioid misuse is a recognized patient safety issue within the medical community. Because many patients are first exposed to opioids after undergoing a surgery and surgeons are amongst the highest prescribers of opioids [2, 3], there has been a push within surgical specialties to educate physicians and their patients on the appropriate use of opioids. These efforts have largely targeted prescribing practices to reduce prescription size without increasing the need for refills due to poor pain management [4, 5]. The Centers for Disease Control suggest early successes from such efforts, estimating a 17% decrease in prescription opioid-involved death rates from 2017 to 2019 [1]. However, recent data demonstrate an increase in overall overdose deaths coinciding with the start of the coronavirus disease (COVID-19) pandemic with over 81,000 deaths occurring in the year ending in May 2020 [6].

While there is some improvement in prescription opioid-related deaths, long-term use of opioids after surgery remains high [2, 3]. To aid surgeons in understanding patients’ risk of chronically using opioids, previous studies have assessed factors that may predispose individuals to opioid abuse after procedures [7, 8]. These studies have suggested younger age, female sex, lower income, and specific medical comorbidities (i.e. pre-surgical opioid use, history of substance abuse, and mental health factors) can predispose patients to prolonged opioid use [9–12]. Knowledge of appropriate opioid use is also likely to play a role. Most literature addressing opioid knowledge has focused on providers’ understanding [13–15]. However, the level of patient understanding regarding opioids and their appropriate use has been minimally studied [16–19]. Specifically, opioid knowledge within the orthopaedic trauma patient population, one of the populations most at risk for opioid misuse [10], has not previously been studied.

A study within an orthopaedic hand surgery specialty practice revealed that while 80% of patients are aware of the addictive properties of opioids, a substantial proportion also possess inaccurate beliefs, such as believing that opioids work well for controlling long-term pain [20]. Given the evidence of existing knowledge gaps, it is essential to identify the specific shortcomings in patient knowledge and to determine what demographic, clinical, or personal experience factors may be associated with misconceptions of opioid use. This information will provide a foundation on which to base future educational interventions.

We therefore sought to assess the degree of understanding regarding opioid use in a consecutive sample of orthopaedic trauma patients and identify knowledge gaps. We also aimed to determine the demographic, clinical, and personal experience factors associated with level of understanding.

## Methods

### Study design and settings

After institutional review board approval, participants were recruited from orthopaedic surgery trauma clinics at two hospital-based clinical sites within our institution. A consecutive sample of all adult patients (≥ 18 years) who visited the identified clinics between August 2009 and November 2020 were invited to participate via an email from the electronic medical record. Informed consent was obtained prior to administering an anonymous 32-question, voluntary online survey with a 7.4 grade Flesch–Kincaid reading level (“Appendix”) [21, 22]. The survey was created using Qualtrics XM (Seattle, WA, USA) and distributed via the MyChart system (Epic Systems Corporation, Verona, WI) to patients meeting our inclusion criteria, 2352 of whom opened the message. The study was performed using the MyChart system, rather than in-person surveys, due to restrictions on in-person research related to the COVID-19 pandemic.

The survey included questions about the following demographic variables: gender, age, race/ethnicity, insurance status, education level, employment status, marital status, and zip code to assess area deprivation index (ADI) quartile [23]. Participants were also asked to answer questions regarding clinical and personal experience with opioid use: chronicity of the injury/accident/problem prompting the clinic visit; whether they underwent surgery; whether they received pain medication prescription, opioid prescription, opioid disposal instructions, or naloxone prescription; whether they were currently using opioids at the time of response, and whether they personally knew someone who has been dependent or overdosed on opioids.

### Study population

Seven percent of patients who opened the message (166 of 2352) completed the survey. Sixty-seven percent of the respondents (111 of 166) identified as female, 31% (52) identified as male, and 2% (3) preferred to not provide their gender (Table 1).

**Table 1:** Characteristics of 166 patients presenting to orthopaedic trauma surgery clinics

Patient characteristics		
Characteristics	No. of respondents	%^a^
Demographics		
*Gender*		
Female	111	67
Male	52	31
No answer	3	1.8
*Age (yrs)*		
18–24	5	3.0
25–34	28	17
35–44	19	11
45–54	33	20
55–64	42	25
65–74	32	19
75–84	3	1.8
85+	1	0.6
No answer	2	1.2
*Race/ethnicity*		
White	136	82
African American	15	9.0
Asian	3	1.8
Hispanic/Latino	5	3.0
Other	3	1.8
No answer	4	2.4
*Insurance status*		
Insured	162	98
Not insured	2	1.2
No answer	2	1.2
*Highest education level*		
High school or less	12	7.2
Some college	14	8.4
2-year degree	6	3.6
4-year degree	49	30
Graduate school or higher	82	49
No answer	3	1.8
*Employment status*		
Full-time	84	51
Part-time	6	3.6
Unemployed	17	10
Retired	35	21
Disabled	14	8.4
Student	4	2.4
No answer	3	1.8
*Marital status*		
Married	96	58
Never married	34	20
Divorced	25	15
Widowed	7	4.2
No answer	4	2.4
*ADI national quartile*		
1	60	36
2	51	31
3	27	16
4	8	4.8
No answer	20	12
Clinical and personal experiences		
*Reason for visit*		
Acute injury/accident/problem < 2 weeks	13	7.8
Subacute injury/accident/problem > 2 weeks	94	57
Chronic problem ≥ 1 year	34	20
No answer	25	15
*Prior surgery*		
Yes	153	92
No	12	7.2
No answer	1	0.6
*Prior pain prescription*		
Yes	164	99
No	1	0.6
No answer	1	0.6
*Prior opioid prescription*		
Yes	156	94
No	4	2.4
No answer	6	3.6
*Current opioid use*		
Yes	18	11
No	138	83
No answer	10	6.0
*Prior receipt of opioid disposal instructions*		
Yes	5	3.0
No	117	70
No answer	44	27
*Prior naloxone prescription*		
Yes	16	9.6
No	140	84
No answer	10	6.0
*Know person—dependent*		
Self	16	9.6
Family	27	16
Friend	35	21
Colleague	2	1.2
Client/student/patient/etc.	4	2.4
None	81	49
No answer	1	0.6
*Know person—overdose*		
Self	2	1.2
Family	12	7.2
Friend	27	16
Colleague	3	1.8
Client/student/patient/etc.	4	2.4
None	117	70
No answer	1	0.6

ADI, Area deprivation index

^a^Percentages may not add to 100 from rounding error

Ninety-four percent of respondents (156) received prior opioid prescription, 10% (16) prior naloxone prescription, and 11% (18) were currently using an opioid. Fifty percent of respondents (84) reported personally knowing someone who has been dependent on opioids, with 19% (16 of 84) reporting self-dependency. Twenty-nine percent of respondents (48) reported knowing someone who had overdosed on opioids, with 4% (2 of 48) reporting self-overdose. Six percent of patients (10) reported knowing both (Table 1).

### Primary and secondary outcomes

Our primary study outcome was to assess trauma patients’ understanding of opioid use. We defined understanding as correct identification and recognition of common opioids, suboxone/subutex, side effects, withdrawal symptoms, and appropriate disposal methods. Secondary outcomes were beliefs regarding time to dependency, safety of using over the counter (OTC) pain medications with opioids, comfort using naloxone, subjective pain level requiring opioid use, harmful effects of opioids, and confidence in their knowledge to safely manage opioid use. We also assessed addiction perception (whether others versus self can become addicted), with a discrepancy meaning they believe that others but not self can become addicted.

### Statistical analysis

Survey data were analysed using descriptive statistics. Associations between demographics and clinical or personal experiences with variables assessing opioid understanding were explored using univariate and multivariable logistic regression (Additional file 1: Table S1, Table 3). The following potential confounders were included in the adjusted model: gender, age, race, marital status, employment status stratified by education level, ADI quartile, injury chronicity, prior opioid prescription, current opioid use, prior naloxone prescription, and knowing someone who has been dependent or addicted on opioids. Analyses were performed using Stata, version 16.0, software (StataCorp LLC, College Station, TX). Alpha was set at 0.05.

## Results

### Current level of patient understanding regarding opioid use

**Fig. 1:**
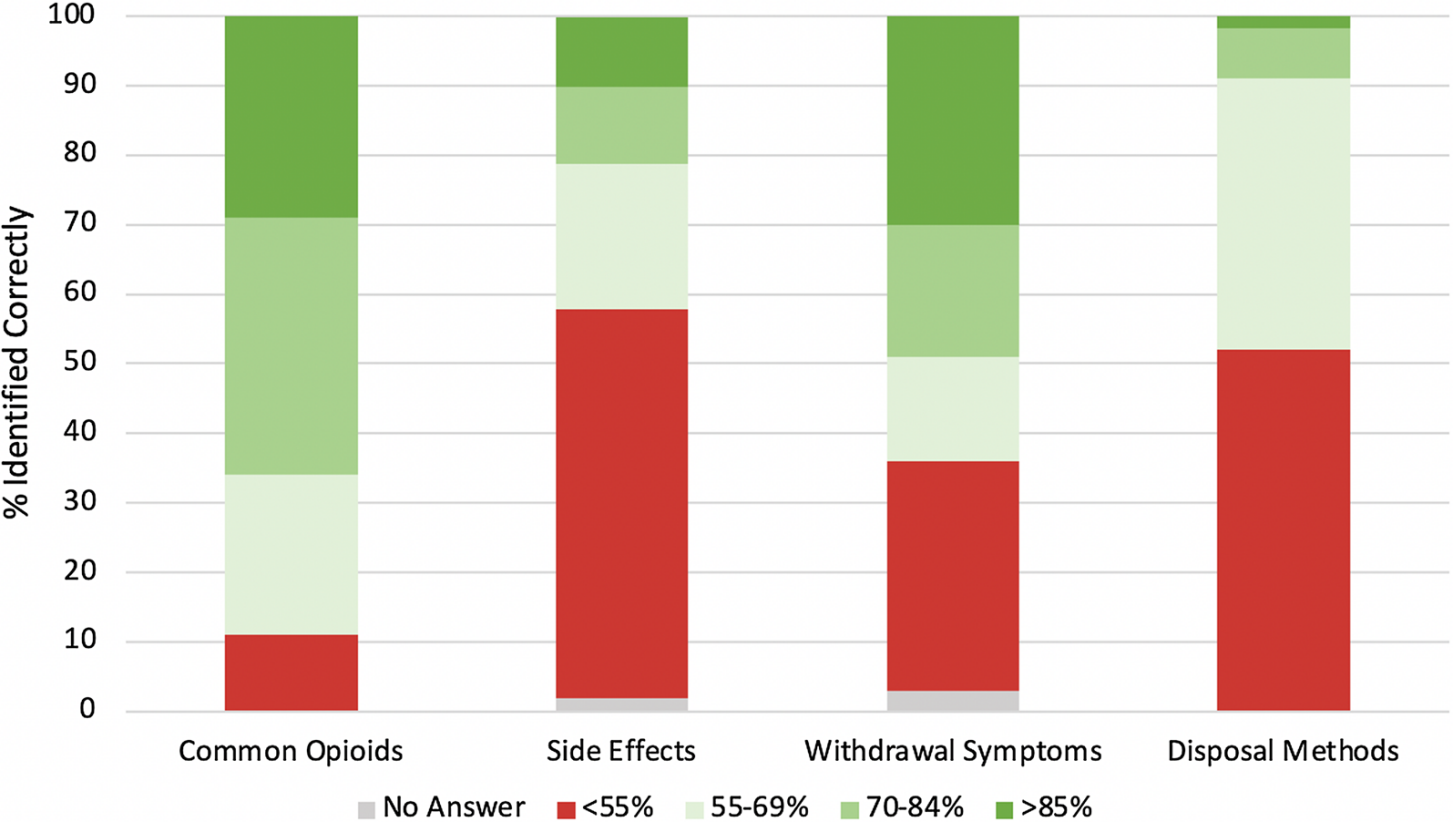
Common opioids, side effects, withdrawal symptoms, and disposal methods identified correctly by orthopaedic trauma patients

Excellent recognition was defined as > 85%, poor as < 55% items identified correctly. Twenty-nine percent of patients (48 of 166) demonstrated excellent recognition of common opioids. Ten percent (17) had excellent recognition of side effects, 30% (50) withdrawal symptoms, and 2.4% (4) appropriate disposal methods. While 11% of patients (18) had poor recognition of common opioids, 56% (93), 33% (54), and 52% (86) poorly recognized side effects, withdrawal symptoms, and disposal methods, respectively (Fig. 1). Further, 34% (56) believed dependence takes weeks to months to develop, and 18% (30) were uncomfortable using naloxone. While patients almost unanimously understood that opioids are harmful and that others can become addicted, 11% (19) had a discrepancy in addiction perception. Almost all patients (88%) stated that they would only take an opioid for moderate to severe pain, defined as a score ≥ 5/10 on the visual analog scale. Eighty-six percent of patients (143) believed they currently know enough to take opioids safely (Table 2).

**Table 2:** Understanding regarding opioid use of 166 patients presenting to orthopaedic trauma surgery clinics

Patient understanding regarding opioid use		
Responses	No. of respondents	%^a^
*1. Identification of opioids*		
85%+ correct	48	29
70–84%	62	37
55–69%	38	23
< 55%	18	11
No answer	0	0.0
*2. Identification of suboxone/subutex*		
Both	24	14
One	26	16
Neither	116	70
No answer	0	0.0
*3. Identification of side effects*		
85%+ correct	17	10
70–84%	18	11
55–69%	35	21
< 55%	93	56
No answer	3	1.8
*4. Identification of withdrawal symptoms*		
85%+ correct	50	30
70–84%	32	19
55–69%	25	15
< 55%	54	33
No answer	5	3.0
*5. Identification of appropriate disposal methods*		
85%+ correct	4	2.4
70–84%	12	7.2
55–69%	64	39
< 55%	86	52
No answer	0	0.0
*6. Time to dependence*		
< 1 week	61	37
1–2 weeks	35	21
2 weeks–1 month	28	17
> 1 month	28	17
No answer	14	8.4
*7. Safety of using OTC pain medication x opioids*		
Yes	59	36
No	60	36
No answer	47	28
*8. Comfort level with using naloxone*		
Extremely comfortable	40	24
Somewhat comfortable	26	16
Neutral	68	41
Somewhat uncomfortable	17	10
Extremely uncomfortable	13	7.8
No answer	2	1.2
*9. Pain level (1–10) requiring opioid use*		
0–2	0	0.0
3–4	3	1.8
5–6	19	11
7–8	75	45
9–10	53	32
No answer	16	9.6
*10. I can become addicted to opioids*		
Yes	140	84
No	19	11
No answer	7	4.2
*11. Others can become addicted to opioids*		
Yes	165	99
No	0	0.0
No answer	1	0.6
*12. Opioids can harm me*		
Yes	159	96
No	5	3.0
No answer	2	1.2
*13. I know enough to take opioids safely*		
Yes	143	86
No	11	6.6
No answer	12	7.2

OTC, Over the counter

^a^Percentages may not add to 100 from rounding error

### Demographics associated with level of understanding

Compared with white patients, non-white patients had 7.8 times greater odds (95% confidence interval [CI] 1.9–31) of signifying a discrepancy in addiction perception (*p* = 0.004). Employment status stratified by education level was also independently associated with knowledge of side effects. Employed patients with the highest education level were less knowledgeable about side effects (adjusted odds ratio [aOR] 0.06, 95% CI 0.006–0.56; *p* = 0.01) and less likely to understand that dependence can occur within 2 weeks of beginning opioids (aOR 0.28, 95% CI 0.09–0.86; *p* = 0.03) than patients who were unemployed. After adjusting for potential confounders, patients in the second least disadvantaged ADI quartile were more knowledgeable about side effects (aOR 8.8, 95% CI 1.7–46) and withdrawal symptoms (aOR 2.7, 95% CI 1.0–7.2; *p* = 0.046) than those in the least disadvantaged quartile. No other demographics were significantly associated with level of patient understanding (Table 3).

**Table 3:** Adjusted (multivariable) odds of orthopaedic trauma surgery patient understanding regarding opioid use

Parameter	> 85% common opioids	Suboxone/subutex	> 85% side effects	> 85% withdrawal symptoms	Dependence < 2 weeks	OTC x opioid safety	Comfort with naloxone	Addiction discrepancy^a^	Know enough to take safely
	Adjusted odds ratio (95% CI)								
*Gender*									
Male^b^	–	–	–	–	–	–	–	–	–
Female	0.68 (0.26–1.8)	0.94 (0.37–2.4)	1.7 (0.38–7.2)	1.1 (0.44–2.7)	2.0 (0.76–5.2)	0.64 (0.22–1.8)	1.7 (0.69–4.2)	0.88 (0.25–3.1)	1.8 (0.28–11)
*Age*									
< 65 years^b^	–	–	–	–	–	–	–	–	–
≥ 65 years	0.38 (0.11–1.3)	0.42 (0.13–1.3)	0.13 (0.01–1.3)	0.63 (0.21–1.9)	1.3 (0.45–4.0)	0.77 (0.24–2.5)	1.1 (0.40–3.0)	1.2 (0.31–4.3)	0.97 (0.11–9.0)
*Race*									
White^b^	–	–	–	–	–	–	–	–	–
Non-white	0.50 (0.12–2.1)	1.1 (0.28–3.9)	1.5 (0.19–11)	1.1 (0.29–4.2)	0.45 (0.13–1.6)	0.57 (0.15–2.1)	1.0 (0.30–3.5)	7.8 (1.9–31)**	1.0
*Marital status*									
Single^b^	–	–	–	–	–	–	–	–	–
Married	0.73 (0.30–1.8)	2.0 (0.79–4.9)	1.6 (0.40–6.2)	1.8 (0.75–4.6)	1.9 (0.79–4.8)	0.81 (0.30–2.2)	0.55 (0.24–1.2)	1.1 (0.35–3.5)	2.7 (0.41–17)
*Employment status stratified by education level*									
Unemployed^b^	–	–	–	–	–	–	–	–	–
Employed w/ < 4-yr degree	1.1 (0.37–3.1)	0.85 (0.30–2.4)	0.67 (0.16–2.8)	0.53 (0.19–1.5)	0.53 (0.19–1.5)	1.4 (0.46–4.1)	1.5 (0.58–3.8)	2.0 (0.55–7.1)	0.53 (0.06–4.5)
Employed w/ ≥ 4-yr degree	1.6 (0.55–4.6)	1.6 (0.55–4.4)	0.06 (0.006–0.56)*	0.77 (0.28–2.2)	0.28 (0.09–0.86)*	0.75 (0.24–2.3)	0.61 (0.22–1.7)	0.73 (0.16–3.4)	1.1 (0.07–17)
*ADI national quartile*									
1^b^	–	–	–	–	–	–	–	–	–
2	0.79 (0.28–2.2)	2.3 (0.86–6.4)	8.8 (1.7–46)**	2.7 (1.0–7.2)*	1.2 (0.43–3.5)	0.51 (0.18–1.5)	1.1 (0.42–2.7)	1.1 (0.32–4.1)	0.13 (0.01–1.5)
3 + 4	0.66 (0.21–2.0)	1.9 (0.62–5.6)	6.1 (0.95–39)	1.5 (0.49–4.4)	1.1 (0.35–3.3)	0.53 (0.16–1.8)	0.65 (0.23–1.9)	0.72 (0.15–3.4)	0.41 (0.02–8.0)
*Chronicity of injury*									
Acute or subacute^b^	–	–	–	–	–	–	–	–	–
Chronic	2.5 (0.79–8.0)	1.3 (0.42–4.0)	0.23 (0.03–1.6)	0.51 (0.15–1.7)	1.0 (0.31–3.4)	0.49 (0.14–1.7)	2.6 (0.89–7.9)	2.7 (0.67–11)	1.0
*Prior opioid prescription*									
No^b^	–	–	–	–	–	–	–	–	–
Yes	1.0	1.0	1.0	1.0	1.0	1.0	1.0	1.0	1.0
*Current opioid use*									
No^b^	–	–	–	–	–	–	–	–	–
Yes	1.9 (0.47–8.0)	0.66 (0.15–2.9)	1.9 (0.47–8.0)	0.27 (0.05–1.6)	0.61 (0.14–2.6)	5.2 (1.0–27)*	1.5 (0.35–6.2)	0.97 (0.16–6.0)	1.0
*Prior naloxone prescription*									
No^b^	–	–	–	–	–	–	–	–	–
Yes	1.3 (0.31–5.2)	1.3 (0.31–5.1)	1.3 (0.31–5.2)	0.73 (0.16–3.3)	0.90 (0.23–3.5)	1.8 (0.42–7.9)	1.8 (0.48–6.9)	0.82 (0.13–5.2)	1.0
*Know someone dependent/overdosed*									
No^b^	–	–	–	–	–	–	–	–	–
Yes	1.9 (0.74–5.0)	2.2 (0.88–5.7)	1.9 (0.74–5.0)	2.6 (1.1–6.5)*	3.8 (1.5–9.8)**	0.78 (0.29–2.1)	1.5 (0.66–3.5)	0.24 (0.07–0.76)*	1.8 (0.26–12)

Bold values indicate significant associations between patient characteristics and measures of understanding regarding opioid use

OTC, Over the counter; ADI, area deprivation index

*Significant at *p* < 0.05

**Significant at *p* < 0.01

***Significant at *p* < 0.001

^a^Addiction discrepancy = patients who believed that others, but not self, can become addicted to opioids

^b^Referent

### Clinical and personal experiences associated with level of understanding

Patients currently using opioids had 5.2 times greater odds (95% CI 1.0–27) of understanding that it is safe to take opioids with OTC pain medications (*p* = 0.046). Patients who knew someone who was dependent or overdosed on opioids were significantly less likely to believe that others but not self can become addicted (aOR 0.24, 95% CI 0.07–0.76; *p* = 0.02). They also possessed greater knowledge regarding withdrawal symptoms (aOR 2.6, 95% CI 1.1–6.5, *p* = 0.03) and were more likely to understand that dependence can develop within 2 weeks of initiating opioids (aOR 3.8, 95% CI 1.5–9.8, *p* = 0.005). No other clinical or personal experiences were significantly associated with level of patient understanding (Table 3).

## Discussion

The safe prescribing of opioids is a critical issue in medicine and especially for orthopaedic trauma surgery. It is crucial to understand and improve the level of patient understanding regarding the use of opioids to improve our care of patients.

Our study illustrates that there are significant gaps in understanding and concerning patterns of beliefs regarding opioid use for orthopaedic trauma surgery patients, even amongst a predominantly high socioeconomic status population. While our respondents had a fairly strong recognition of common opioids, they had a poor understanding of side effects, withdrawal symptoms, and appropriate disposal methods. One potential explanation may be that commercial media has improved public recognition of common opioids, but awareness of their medical implications is still lacking [24, 25] This theory is further supported by our results illustrating greater identification of side effects, such as addiction (87%) and constipation (78%), that have received more media attention [26].

Our study demonstrates the need for additional education of all our patients in several areas. A subset of the respondents believe that they cannot become addicted to opioids, even when they recognize the addictive potential for others. This is consistent with the results of previous studies, which have shown inaccurate perception of individual risk may be due to addiction stigma or the perception that addiction is a moral failing that can be resisted through personal willpower [27–29]. Patients also lacked knowledge about the rapid nature of opioid dependence and naloxone use [30, 31]. Patients had a better understanding of dependence and appropriate dosing, as over half of our patients understand that dependence can occur within 2 weeks and patients almost universally would not take an opioid for mild pain levels. This may demonstrate early successes with patient education on opioid use.

We found that race was independently associated with level of understanding, with patients identifying as white being significantly less likely to have discrepancy in addiction perception [32, 33]. This may be attributed to an awareness that the most dramatic increase in overdose mortality in the last two decades occurred amongst non-Hispanic whites [34, 35]. Much of the recent social and political attention on the opioid epidemic has also been focused on this population [36, 37]. While use amongst whites has been framed as a public health crisis, the media has traditionally portrayed blacks with opioid use disorders as having a moral shortcoming and undergoing greater criminalization [36]. Minorities thus may have alternative perceptions regarding opioid use and self-addiction. However, our findings cannot be confidently interpreted without more robust quantitative studies that do not use race as a surrogate for other factors (i.e., health behaviours).

We further illustrated that employment with higher education level and being from the least disadvantaged ADI quartile were associated with lesser levels of knowledge regarding opioid use when controlling for potential confounders. This contrasts with the results of Bargon et al. [20], who demonstrated higher education to be associated with patients understanding the importance of opioid prescribing policies, suggesting greater knowledge of opioid misuse. This conflicting and limited evidence emphasizes the necessity for further studies evaluating patient knowledge of opioids in orthopaedic surgery. Our findings may reflect that patients of lower socioeconomic measures and from more disadvantaged geographic regions are more frequently exposed to opioid prescription, chronic consumption, and related overdose in their communities [38–41]. This exposure, in turn, may translate to greater knowledge regarding opioids.

In addition, patients had significantly greater levels of understanding if they were currently using opioids or knew someone who misused opioids. This aligns with the results of Razdan et al., who determined that knowledge regarding opioid use for paediatric patients was greater for parents who knew someone who had become addicted to opioids [42]. Stover et al. [43] also demonstrated that young adults who received prior naloxone training or opioid prescriptions scored significantly higher on the Opioid Overdose Knowledge Scale. It is possible that these patients were educated by their prescribing physician or distributing pharmacist on the appropriate use and risks involved. Another plausible explanation is that in light of the ongoing opioid epidemic, patients were more aware of the intrinsic risks and more inclined to research these risks upon receiving prescriptions or having someone they know become dependent or overdosing on opioids [43]. Recent initiatives to educate patients through counseling and public materials thus may be effective, and it is important for surgeons to follow recommendations for co-prescription of naloxone and opioids. Altogether, these results suggest that clinical and personal experience with opioids should be emphasized when taking patient histories, and those with positive histories may be more knowledgeable in the appropriate use and risks of taking prescription opioids in the postoperative period.

Our study is not without limitations. The electronic format of our survey distribution was a result of the COVID-19 pandemic, which restricted access to patient care areas (i.e. trauma clinics). Subsequently, the study was limited by responder bias with the low response rate. Responders likely differ from non-responders, especially since the survey’s electronic format tends to attract more white responders possessing higher socioeconomic measures [44, 45]. As a result, the respondents did not accurately represent the trauma patient population seen by our institution, which serves a clinically diverse, minority-majority patient population within an urban environment. However, it must be noted that the response rate was based on the 2352 patients who opened the survey invitation, thereby controlling for internet access and associated socioeconomics to an extent. Respondents also likely had more personal experiences with opioids which motivated them to answer the survey. Despite the high socioeconomic measures of our cohort, we were able to capture 17 unique individuals (10%) who reported self-dependency or overdose on opioids, which is higher than the estimated national rate of prescription opioid misuse (3.6%) amongst people aged 12 years or older [46]. Overall, our respondent characteristics would trend toward greater awareness of opioids and the importance of understanding their appropriate use [20, 43], so the low level of understanding in our results likely overestimates opioid knowledge, emphasizing the importance of presenting our findings.

## Conclusions

In conclusion, all prescribers should be aware that there is a low level of understanding regarding opioid use in patients who present to orthopaedic trauma surgery clinics. This places patients at higher risk for misusing opioids if prescribed. It is essential to emphasize that providers should not exacerbate existing prescribing disparities and reduce access to opioids for patients with lesser understanding. Rather, more involved educational interventions should be taken with these patients to mediate risk for misuse, and improvements in overall patient education should be established at all institutions. To more comprehensively study patient understanding, future studies should apply larger scale, multidisciplinary, multicenter surveys and investigate the effectiveness of specific educational interventions in improving patient knowledge. Application of robust qualitative methods grounded in well-established social theories, like the Health Belief Model, which is outside the scope of this study, would be better able to provide reasons behind the perceptions assessed here as well.

### Supplementary Information

**Additional file 1**. Unadjusted (univariate) odds of orthopaedic trauma surgery patient understanding regarding opioid use.

## Data Availability

The datasets used and/or analysed during the current study are available from the corresponding author on reasonable request.

## Appendix

### Survey on trauma patient knowledge and perspectives about opioids

#### Medication knowledge

1. An opioid is a specific type of prescribed pain medication. Which of the following which are opioids? Select all that apply.
  (a) Ibuprofen, (b) Aspirin, (c) Morphine, (d) Methadone, (e) Codeine, (f) Oxycodone, (g) Hydrocodone, (h) Hydromorphone, (i) Oxycontin, (j) Percocet, (k) Dilaudid, (l) Tylenol #3, (m) Gabapentin, (n) Fentanyl, (o) Heroin, (p) Tramadol, (q) Caffeine, (r) Steroids, (s) Buprenorphine, (t) Naloxone, (u) Subutex, (v) Suboxone
2. Side effects are undesirable effects of a drug or treatment. Which of the following are side effects of taking opioids? Select all that apply.
  (a) Constipation, (b) Nausea, (c) Fatigue, (d) Mental fog, (e) Liver failure, (f) Dizziness, (g) Gastrointestinal bleeding, (h) Vomiting, (i) Trouble breathing, (j) Skin rash, (k) Increased pain, (l) Depression, (m) Addiction, (n) Cancer, (o) Death
3. Opioid withdrawal is when a person experiences discomfort when a medication is no longer taken regularly. Which of the following are signs/symptoms of opioid withdrawal? Select all that apply.
  (a) Paranoia, (b) Nausea, (c) Depression, (d) Chills, (e) Tremors, (f) Sweating, (g) Itching, (h) Constipation, (i) Poor coordination, (j) Restlessness, (k) Anxiety
4. Dependence is the body’s physical and mental need for continued opioid use. How long do you think it takes to develop opioid dependence?
  (a) Less than 1 week, (b) More than 1 week but less than 2 weeks, (c) More than 2 weeks but less than 1 month, (d) More than 1 month but less than 3 months, (e) More than 3 months but less than 6 months, (f) More than 6 months, (g) Other
5. Is it safe to take over-the-counter pain medications at the same time as an opioid?
  (a) Yes, (b) No, (c) Don’t know
6. What are acceptable ways to dispose of unused opioid pain medications? Select all that apply.
  (a) Pour it down the sink, (b) Flush it down the toilet, (c) Throw away pills in household trash, (d) Return to the police, (e) Return to the pharmacy, (f) Return to a “take back” program, (g) Keep it for future use for self, (h) Keep for future use of a family member or friend, (i) Other

#### Personal experience

(7) Do you know or have you known someone who had ever been dependent on prescribed pain medication?
  (a) No, (b) Self, (c) Partner, (d) Family member, (e) Friend, (f) Other, (g) Don’t know
(8) Do you know or have you known someone who had ever had an overdose from prescribed pain medication or a pain medication obtained through other means?
  (a) No, (b) Self, (c) Partner, (d) Family member, (e) Friend, (f) Other, (g) Don’t know
(9) How comfortable are you with using naloxone in the event of an overdose?
  (a) Extremely comfortable, (b) Somewhat comfortable, (c) Neither comfortable nor uncomfortable, (d) Somewhat uncomfortable, (e) Extremely uncomfortable
(10) Have you had a prior surgery?
  (a) Yes, (b) No
(11) Have you ever been prescribed medication for pain?
  (a) Yes, (b) No, (c) Don’t know
(12) [If “yes” to 11] Have you ever been prescribed an opioid medication for pain?
  (a) Yes, (b) No, (c) Don’t know
(13) [If “yes” to 12] Are you currently taking an opioid medication for pain?
  (a) Yes, (b) No, (c) Don’t know
(14) [If “yes” to 12] Have you ever been prescribed naloxone to use in the event of an overdose?
  (a) Yes, (b) No, (c) Don’t know
(15) [If “yes” to 12] Did you receive instructions on proper opioid disposal with your prescription?
  (a) Yes, (b) No, (c) Don’t know

#### Beliefs and perspectives

(16) Do you think that opioids can harm you?
  (a) Yes, (b) No, (c) Don’t know
(17) Do you think that you can get addicted to taking opioids?
  (a) Yes, (b) No, (c) Don’t know
(18) Do you think that other people can get addicted to taking opioids?
  (a) Yes, (b) No, (c) Don’t know
(19) At what level of pain would you take an opioid for pain control on a scale of 0–10, with 0 meaning no pain and 10 meaning the worst pain?
  [respond on 0–10 scale]
(20) Do you think you know what you need to take opioids safely?
  (a) Yes, (b) No, (c) Don’t know
(21) Would you appreciate more information on opioids?
  (a) Yes, (b) No
(22) What are 1–5 words that come to mind when you think about opioids?
  [open response]
(23) What else should we know? Please do NOT include any information which could be used to identify you.
  [open response]

#### Patient characteristics

(24) Gender
  (a) Male, (b) Female, (c) Other
(25) Age (years)
  (a) Under 18, (b) 18–24, (c) 25–34, (d) 35–44, (e) 45–54, (f) 55–64, (g) 65–74, (h) 75–84, (i) 85 or older
(26) Race/ethnicity
  (a) White, (b) Black or African American, (c) Hispanic or Latino, (d) Asian, (e) American Indian or Native Alaskan, (f) Native Hawaiian or Pacific Islander, (g) Other
(27) Insurance status
  (a) Insured, (b) Uninsured, (c) Don’t know
(28) Employment
  (a) Employed full time, (b) Employed part time, (c) Unemployed looking for work, (d) Unemployed not looking for work, (e) Retired, (f) Student, (g) Disabled
(29) Marital status
  (a) Married, (b) Widowed, (c) Divorced, (d) Separated, (e) Never married
(30) Zip code
  [open response]
(31) Highest level of education
  (a) Less than high school, (b) High school graduate, (c) Some college, (d) 2-year degree, (e) 4-year degree, (f) Professional degree, g) Doctorate
(32) Reason for clinic visit
  (a) Injury/accident/problem that started less than 2 weeks ago, (b) Injury/accident/problem that started more than 2 weeks ago, (c) Chronic problem/pain for at least a year

## Abbreviations

COVID-19: Coronavirus disease
ADI: Area deprivation index
OTC: Over the counter
