# Supplementary material for: Patient understanding regarding opioid use in an orthopaedic trauma surgery population: a survey study
Source: J Orthop Surg Res. 2021 Dec 24;16:736. doi: 10.1186/s13018-021-02881-w (PMC8709537; doi:10.1186/s13018-021-02881-w)
Supplement: Supplementary file 1 — Additional file 1. Unadjusted (univariate) odds of orthopaedic trauma surgery patient understanding regarding opioid use. [file 13018_2021_2881_MOESM1_ESM.docx]

**Supplementary Table 1** Unadjusted (Univariate) Odds of Orthopaedic Trauma Surgery Patient Understanding Regarding Opioid Use

| **Parameter** | **>85% Common Opioids** | **Suboxone/**  **Subutex** | **>85% Side Effects** | **>85% Withdrawal Symptoms** | **Dependence <2 Weeks** | **OTC x Opioid Safety** | **Comfort with Naloxone** | **Addiction Discrepancy**‡ | **Know Enough to Take Safely** |
| --- | --- | --- | --- | --- | --- | --- | --- | --- | --- |
|  | **Unadjusted Odds Ratio (95% Confidence Interval)** | | | | | | | | |
| *Gender* | | | | | | | | | |
| Male† | - | - | - | - | - | - | - | - | - |
| Female | 0.91 (0.47–1.8) | 0.77 (0.38–1.5) | 0.97 (0.83–1.1) | 0.96 (0.84–1.1) | 1.0 (0.98–1.0) | 1.0 (0.94–1.1) | 1.0 (0.98–1.0) | 1.0 (0.99–1.0) | 1.8 (0.52–6.1) |
| *Age* | | | | | | | | | |
| <65 years† | - | - | - | - | - | - | - | - | - |
| ≥65 years | 0.49 (0.20–1.2) | 0.46 (0.19–1.1) | 0.20 (0.03–1.6) | 0.50 (0.20–1.2) | 1.1 (0.49–2.4) | 0.63 (0.25–1.5) | 1.1 (0.50–2.3) | 1.2 (0.43–3.2) | 0.71 (0.18–2.9) |
| *Race* | | | | | | | | | |
| White† | - | - | - | - | - | - | - | - | - |
| Non-white | 0.67 (0.25–1.8) | 1.2 (0.51–3.0) | 0.66 (0.14–3.1) | 0.48 (0.17–1.3) | 0.32 (0.13–0.78) | **0.34 (0.12–0.95)*** | 0.72 (0.30–1.7) | **5.4 (2.1–14)***** | 0.82 (0.16–4.1) |
| *Marital status* | | | | | | | | | |
| Single† | - | - | - | - | - | - | - | - | - |
| Married | 1.0 (0.97–1.0) | 0.95 (0.82–1.1) | 0.94 (0.57–1.6) | 0.97 (0.88–1.1) | 0.99 (0.97–1.0) | 0.99 (0.97–1.0) | 1.0 (0.97–1.0) | 1.0 (0.99–1.0) | 1.0 (0.81–1.3) |
| *Employment status stratified by education level* | | | | | | | | | |
| Unemployed† | - | - | - | - | - | - | - | - | - |
| Employed w/  < 4-yr degree | 0.63 (0.27–1.5) | 0.61 (0.27–1.4) | 0.51 (0.15–1.7) | 0.54 (0.24–1.2) | 0.49 (0.22–1.1) | 1.5 (0.63–3.5) | 0.94 (0.45–1.9) | 1.3 (0.52–3.5) | 0.62 (0.16–2.5) |
| Employed w/  ≥ 4-yr degree | 1.5 (0.68–3.5) | 1.3 (0.60–3.0) | **0.09 (0.01–0.70)*** | 1.0 (0.44–2.3) | 0.45 (0.19–1.1) | 0.93 (0.38–2.3) | 0.95 (0.43–2.1) | 0.63 (0.18–2.1) | 2.4 (0.26–23) |
| *ADI national quartile* | | | | | | | | | |
| 1† | - | - | - | - | - | - | - | - | - |
| 2 | 0.88 (0.39–2.0) | 1.6 (0.72–3.7) | 2.7 (0.75–9.4) | 1.7 (0.77–3.9) | 1.4 (0.61–3.1) | 0.63 (0.26–1.5) | 1.3 (0.62–2.9) | 1.4 (0.51–3.7) | 0.62 (0.13–2.9) |
| 3 + 4 | 0.69 (0.26–1.8) | 1.2 (0.47–3.1) | 1.8 (0.42–7.7) | 0.99 (0.38–2.6) | 1.0 (0.42–2.5) | 0.69 (0.26–1.8) | 1.4 (0.59–3.3) | 0.94 (0.29–3.1) | 0.85 (0.13–5.4) |
| *Chronicity of Injury* | | | | | | | | | |
| Acute or subacute† | - | - | - | - | - | - | - | - | - |
| Chronic | 2.0 (0.88–4.4) | 1.3 (0.57–2.9) | 0.72 (0.19–2.7) | 0.84 (0.36–2.0) | 1.2 (0.51–2.9) | 0.67 (0.28–1.6) | **2.9 (1.3–6.6)**** | 2.2 (0.83–5.9) | 2.8 (0.34–23) |
| *Prior opioid prescription* | | | | | | | | | |
| No† | - | - | - | - | - | - | - | - | - |
| Yes | 1.2 (0.12–12) | 1.3 (0.13–13) | 1.0 | 1.0 | 1.7 (0.24–13) | 1.0 | 0.67 (0.09–4.9) | 1.0 | 7.7 (0.63–93) |
| *Current opioid use* | | | | | | | | | |
| No† | - | - | - | - | - | - | - | - | - |
| Yes | **2.8 (1.0–7.7)*** | 1.2 (0.42–3.4) | 1.0 (0.21–4.9) | 0.84 (0.28–2.5) | 1.1 (0.37–3.1) | 3.2 (0.94–11) | **3.5 (1.2–9.8)*** | 1.1 (0.28–4.0) | 1.0 |
| *Prior naloxone prescription* | | | | | | | | | |
| No† | - | - | - | - | - | - | - | - | - |
| Yes | 2.8 (0.97–8.0) | 1.4 (0.49–4.2) | 1.2 (0.24–5.7) | 0.66 (0.20–2.2) | 0.96 (0.33–2.8) | 2.2 (0.70–6.9) | **3.8 (1.2–12)*** | 0.73 (0.15–3.4) | 1.0 |
| *Know someone dependent/overdosed* | | | | | | | | | |
| No† | - | - | - | - | - | - | - | - | - |
| Yes | **1.6 (1.2–2.1)**** | **1.4 (1.1–1.9)*** | **1.8 (1.2–2.8)**** | 1.3 (0.99–1.7) | **1.4 (1.1–2.0)*** | 1.1 (0.84–1.5) | 1.2 (0.93–1.6) | **0.61 (0.39–0.96)*** | 1.2 (0.68–2.1) |

OTC, over the counter; ADI, area deprivation index

‡Addiction discrepancy = patients who believed that others, but not self, can become addicted to opioids.

†Referent

*Significant at p<0.05

**Significant at p<0.01

***Significant at p<0.001
